# Mechanisms and targets of harnessing *Culex pipiens*-specific antibodies as a novel vector control strategy

**DOI:** 10.7555/JBR.39.20250135

**Published:** 2026-03-19

**Authors:** Xuebin Zhao, Jian Zheng, Weimin Zheng, Jinrong Lin, Guangshuo Ding, Xinhui Yu, Jun Cao, Yun Chen, Bo Shen, Gaoqian Feng

**Affiliations:** 1 National Vaccine Innovation Platform, NHC Key Laboratory of Antibody Technique, Jiangsu Key Laboratory of Pathogen Biology, Department of Pathogen Biology, Nanjing Medical University, Nanjing, Jiangsu 211166, China; 2 Key Laboratory of National Health Commission on Parasitic Disease Control and Prevention, Key Laboratory of Jiangsu Province on Parasite and Vector Control Technology, Jiangsu Institute of Parasitic Diseases, Wuxi, Jiangsu 214064, China; 3 The Affiliated Taizhou People's Hospital of Nanjing Medical University, Taizhou School of Clinical Medicine, Nanjing Medical University, Taizhou, Jiangsu 225300, China; 4 Jiangsu Key Laboratory of Pathogen Biology, Department of Pathogenic Biology, Nanjing Medical University, Nanjing, Jiangsu 211166, China; 5 State Key Laboratory of Reproductive Medicine and Offspring Health, School of Pharmacy, Nanjing Medical University, Nanjing, Jiangsu 211166, China

**Keywords:** *Culex*, antibodies, classical complement pathway, oviposition, mosquito control

## Abstract

Mosquito-borne diseases pose a significant global health threat, necessitating the development of innovative vector control strategies. In this study, we investigated the potential of harnessing host immunity against mosquitoes through vaccination. Using *Culex pipiens* (*C. pipiens*) as a model, we demonstrated that polyclonal antibodies against *C. pipiens* abdominal protein extracts significantly impaired oviposition and increased mosquito mortality, primarily through the classical complement activation pathways. However, repeated exposure led to resistance, indicating potential adaptation. Proteomic analysis identified metabolic proteins as key targets, with Gene Ontology and Kyoto Encyclopedia of Genes and Genomes enrichment analyses highlighting their roles in carboxylic acid metabolism, tyrosine degradation, and the proteasome pathways. Notably, cross-species reactivity was revealed by Western blotting, showing strong binding of *Culex*-specific antibodies to *Anopheles* and *Aedes* abdominal proteins. This study provides mechanistic insights into antibody-based mosquito suppression, highlighting its potential as an innovative vector control strategy while underscoring the need for further research on resistance management and ecological impacts.

## Introduction

Mosquito-borne diseases remain a persistent global health challenge, with *Culex pipiens* (*C. pipiens*) serving as a key vector for pathogens, including Japanese encephalitis virus and filarial nematodes^[1–2]^. Therefore, controlling mosquito vectors remains the focus of blocking the transmission of multiple mosquito-borne diseases^[3]^. However, despite extensive efforts to control mosquito populations through insecticides and environmental management, the emergence of insecticide resistance and ecological concerns underscore the urgent need for innovative, targeted strategies^[4]^. Early studies have explored the potential of vaccine-based interventions to disrupt mosquito physiology, leveraging the host immune components in the blood meal to impair the survival or reproduction of mosquitoes^[5–6]^. However, due to technical limitations at the time, little progress was made beyond the initial observations. Thus, a detailed understanding of the mechanisms and targets of the antibodies is required for further development of such a technique into a practical approach.

Previous studies have demonstrated that polyclonal antibodies targeting critical mosquito antigens may compromise mosquito fitness and reproductive capacity^[7–10]^. One proposed mechanism is that host antibodies and other immune components are carried along with the blood meal and become activated in the mosquito midgut, causing tissue damage. A vaccine against ticks and tick-borne diseases was developed based on a similar mechanism^[11]^. Furthermore, antibodies against *Anopheles* midgut proteins have shown promise in reducing malaria transmission by interrupting the interaction between the ookinetes and the mosquito midgut^[12]^, suggesting that antibody-complement-dependent cytotoxicity can be harnessed against disease vectors. Nevertheless, these efforts have been limited by variable cross-reactivity across species, incomplete knowledge of antigen specificity, and the lack of mechanistic insights into how antibodies exert their effects *in vivo*. Furthermore, the functional consequences of targeting distinct anatomical regions—such as the abdomen (encompassing reproductive and metabolic tissues) versus the midgut—remain poorly characterized.

In the present study, we conducted a proof-of-concept investigation to explore the potential of developing a transmission-blocking vaccine by targeting mosquito vectors. We hypothesized that antibodies raised against *C. pipiens* antigens could impair mosquito survival and reproduction through tissue-specific mechanisms and also exhibit cross-reactivity with other medically relevant mosquito species. To test our hypothesis, we harnessed current understanding of antibody functions and advanced proteomics approaches. This integrated approach not only provided mechanistic insights into antibody-mediated mosquito suppression but also identified metabolic pathways as potential targets for vector control.

## Materials and methods

### Main reagents and equipment

Insect tissue protein extraction kit (Invent Biotechnologies, Eden Prairie, MN, USA), Bicinchoninic Acid (BCA) protein assay kit (Thermo Fisher Scientific, Waltham, MA, USA), polyvinylidene fluoride (PVDF) membrane (Millipore, Bedford, MA, USA), enhanced chemiluminescence substrate (Thermo Fisher Scientific), Glycogen Periodic Acid-Schiff (PAS) Stain Kit (Solarbio, Beijing, China), complement component 9 (C9) antibody (1∶1000, Cat. #MA5-33373, Thermo Fisher Scientific), goat anti-rabbit secondary antibody (1∶500, Cat. #YFSA02, Yifeixue Biotechnology, Nanjing, Jiangsu, China), complement component 1q (C1q)-FITC antibody (1∶1000, Cat. #0855166, MP Biomedicals, Santa Ana, CA, USA), and Pierce Crosslink Magnetic immunoprecipitation (IP)/co-immunoprecipitation (Co-IP) Kit (Thermo Fisher Scientific).

Multi-functional enzyme-linked immunosorbent assay reader (BioTek Instruments, Winooski, VT, USA), affinity chromatography protein purifier (Cytiva, Uppsala, Sweden), centrifuge (Thermo Fisher Scientific), artificial climate incubator (LISK, Nanjing, Jiangsu, China), Western blotting (WB) detection system (Bio-Rad Laboratories, Hercules, CA, USA), ultra-high sensitivity chemiluminescence imaging system (Bio-Rad Laboratories), stereomicroscope (CEWEI, Shanghai, China), optical microscope (CEWEI), laser microscope (Zeiss, Oberkochen, Germany), and quadrupole time-of-flight mass spectrometer (Bruker, Billerica, MA, USA).

### Mosquito rearing

*Culex pipiens pallens* (hereafter referred to *C. pipiens*), *Anopheles sinensis*, and *Aedes aegypti* were reared in incubators at 26–28 ℃, 80% humidity, and a daily light cycle of 12 h with a light intensity of 2000 lumens. The larvae were fed a mixture of porcine liver powder, yeast powder, and fish feed. BALB/c mice were anesthetized *via* tail-vein injection of an equal mixture of xylazine hydrochloride and tiletamine-zolazepam hydrochloride to provide blood meals for adult mosquitoes twice a week. At other times, a 10% glucose solution was provided.

### Preparation of mosquito antigens

Female *C. pipiens* that had fed on fresh blood were killed by freezing at −40 ℃. The intersegmental membranes of the thorax or abdomen were punctured with a fine needle, and a capillary tube was used to collect the outflowing hemolymph while avoiding contamination. Then, the abdomen was cut open using fine forceps and a micro-needle to separate the midgut. The midguts were rinsed repeatedly in PBS to remove ingested blood. Subsequently, crude protein extraction from the mosquito abdomen and midgut was performed using an insect tissue protein extraction kit according to the manufacturer's instructions. Protein concentration was determined using the BCA protein assay kit.

### Preparation of antibodies

New Zealand white rabbits (*Oryctolagus cuniculus*) were immunized with protein extracts from the abdomen, midgut, or hemolymph collected from *C. pipiens* to generate polyclonal antibodies, using Freund's adjuvant to enhance immune responses. After the immunization was completed, peripheral blood was collected from the rabbits and centrifuged at 4 000 *g* for 10 min at 4 ℃ to obtain the serum, which was then stored at −80 ℃. The IgG antibodies from the rabbit serum were purified using an AKTA pure protein purification system with Protein A resin. The purified antibodies were concentrated using a 100 kDa ultrafiltration tube by centrifugation at 3 000 *g* for 40 min. The volume of the antibody solution was reduced to one-ninth of the original serum volume, achieving a nine-fold concentration. The concentration of these antibodies was determined using the BCA method, and the antibodies were stored at −80 ℃ for subsequent membrane feeding experiments.

For generating *C. pipiens* abdomen-specific antibodies, mice were immunized *via* subcutaneous injection with whole abdomen protein and Freund's adjuvant. These antibodies were subsequently used in experiments to inhibit the reproduction of *C. pipiens* offspring.

### Enzyme-linked immunosorbent assay (ELISA)

To detect antibody binding to the abdomen, midgut, and hemolymph protein extracts, the protein extracts from *C. pipiens* abdomen, midgut, and hemolymph were diluted in sodium carbonate-sodium bicarbonate buffer (pH 9.6). These diluted proteins were then coated onto a 96-well microplate (50 μL/well) and incubated overnight at 4 ℃. After washing the 96-well microplate, 200 μL of 1% casein solution was added to each well and incubated at 37 ℃ for 2 h, followed by another wash. The antibodies from the abdomen, midgut, hemolymph, and negative serum were serially diluted twofold using 0.1% casein, and 50 μL of each dilution was added to the wells of the 96-well microplate. The last well received only an equal volume of 0.1% casein as a blank control. The plate was then incubated at room temperature for 1 h. After washing, 50 μL of a 1∶2 500 dilution of goat anti-rabbit HRP-conjugated antibody was added to each well and incubated at room temperature for 1 h. Following another wash, 50 μL of ABTS substrate was added to each well and incubated for 10–20 min for color development. The reaction was then stopped by adding 50 μL of 1% SDS to each well. The optical density at 405 nm (OD_405_) was measured using a microplate reader.

### WB analysis

To further analyze the binding characteristics of antibodies from the abdomen, midgut, and hemolymph to their respective antigens, as well as to antigenic proteins from other mosquito species, WB analysis was conducted. The extracted proteins from the abdomen, midgut, thorax, and head of *C. pipiens*, as well as the abdominal proteins of *Anopheles sinensis* and *Aedes aegypti*, were quantified using the BCA method to determine protein concentrations. Subsequently, the proteins were denatured in SDS-PAGE gel loading buffer at 98 ℃ for 10 min. A total of 20 μg of protein was loaded onto a 12% SDS-polyacrylamide gel and then transferred onto a PVDF membrane. The membrane was blocked at room temperature in blocking buffer containing 1% casein for 2 h, followed by incubation with antibodies against the abdomen, midgut, and hemolymph at 4 ℃ overnight. After washing, the membrane was incubated with horseradish peroxidase (HRP)-conjugated goat anti-rabbit IgG secondary antibody at room temperature for 2 h. The ECL substrate was applied to the membrane for 30 s, and protein bands were visualized using a WB detection system.

### Assessment of mosquito mortality and oviposition by membrane feeding assay

Female mosquitoes aged 5–7 days were starved for 48 h, during which only water was provided. The mosquitoes were transferred into small paper cylinders, with the top openings sealed with nylon mesh. The lower opening of the membrane feeder was sealed with a Parafilm membrane that had been wiped with sweat. The glass apparatus was then placed on top of the paper cylinder, ensuring that the Parafilm faced the nylon mesh of the cylinder. The outer chamber of the glass apparatus was filled with 37 ℃ warm water, while the inner chamber contained a mixture of antibodies and fresh human blood at ratios of 1∶9 (high concentration) or 1∶99 (low concentration). After 3 h, most blood-fed mosquitoes were engorged. They were then briefly anesthetized at 4 ℃ for 3–4 min and immediately sorted into 50 mL centrifuge tubes, with one mosquito per tube. The tube openings were sealed with nylon mesh, and the mosquitoes were provided with a 10% glucose solution for nutrition. On the following day, once the mosquitoes had largely regained their flying ability, approximately 1 mL of ultrapure water was added to each tube to provide an oviposition site. Daily observations were made to record mosquito mortality and oviposition. When mosquitoes laid eggs, they were transferred to new 50 mL centrifuge tubes. The egg rafts from the original tubes were transferred onto filter paper moistened with ultrapure water, and photographed and counted under a stereomicroscope.

### Detection of antibody effects on mosquitoes and assessment of offspring's reproduction

Mice were passively immunized with whole abdomen-specific mouse antibodies prior to providing a blood meal to mosquitoes. Mosquito colonies were continuously maintained, and oviposition was monitored across generations F5 to F7.

### Histological observation

Mosquitoes were fixed in Bouin's solution for 24 h and then embedded in paraffin. Sections of 5 μm thickness were cut, dewaxed in xylene, rehydrated through a graded series of ethanol, and routinely stained with hematoxylin and eosin (H&E). Images were captured using an optical microscope.

For glycogen detection, rehydrated sections were processed according to the manufacturer's instructions using the Glycogen PAS Stain Kit, and images were captured using an optical microscope.

For immunohistochemistry, rehydrated sections were subjected to antigen retrieval in citrate buffer (pH 6.0) at 95 ℃ for 30 min. Then, the sections were treated with 0.3% H_2_O_2_ in PBS for 10 min to quench endogenous peroxidase activity and were washed in PBS. The slides were blocked with 5% bovine serum albumin (BSA) for 1 h, followed by incubation with antibodies against the abdomen, midgut, hemolymph, and C9 in a humid chamber at 4 ℃ overnight. Subsequently, the sections were incubated with biotinylated goat anti-rabbit secondary antibody for 1 h and treated with streptavidin-biotin complex for 1 h. Positive cells were visualized by treatment with diaminobenzidine. Corresponding isotype controls were used as negative controls. The sections were counterstained with hematoxylin, and images were captured using an optical microscope.

For immunofluorescence, following antigen retrieval, the sections were blocked with 5% BSA for 1 h, followed by overnight incubation with C1q-FITC in a humid chamber at 4 ℃. After washing in PBS, the sections were counterstained with 4',6-diamidino-2-phenylindole for 5 min and observed under a confocal laser microscope.

### IP and sodium dodecyl sulfate-polyacrylamide gel electrophoresis (SDS-PAGE)

Co-IP was performed using the Pierce Crosslink Magnetic IP/Co-IP Kit following the manufacturer's instructions. Briefly, 0.8–1.4 μg of whole abdomen-specific antibodies and negative control antibodies were coated onto magnetic beads and then incubated with crude protein extracts from the midgut and abdomen of blood-fed female *Anopheles* mosquitoes at room temperature for 3 h with rotation. Subsequently, 200 μL of elution buffer was added, and the mixture was incubated at room temperature for 10 min with rotation. Magnetic beads were collected using a magnet, and the supernatant was collected and lyophilized. Six samples were obtained, including three from the experimental group and three from the control group. Each sample was resuspended in 20 μL of 0.5 mol/L dithiothreitol and mixed thoroughly until the solution became clear. Then, 10 μL of each sample was taken and mixed with loading buffer. Samples were heated in a metal bath and separated using ACE precast gels. The gel was stained using Coomassie Brilliant Blue G-250. Gel bands of interest were excised and transferred to tubes.

### In-gel digestion and liquid chromatography-tandem mass spectrometry (LC-MS/MS) analysis

Gel pieces were destained with a 50% acetonitrile solution, reduced with 10 mmol/L tris (2-carboxyethyl) phosphine and 20 mmol/L chloroacetamide at 37 ℃ for 60 min, and then dried twice with acetonitrile. The dried gels were rehydrated with trypsin solution (20 ng/nL in 25 mmol/L NH_4_HCO_3_) and incubated at 37 ℃ for 15 h. The peptides from each lane were extracted from the gel twice with 50% acetonitrile/0.1% trifluoroacetic acid for 30 min. Then the peptide mixture was desalted using C18 ZipTips according to the manufacturer's instructions.

The extracted solution was concentrated using a SpeedVac and then resuspended with 8 μL of solvent A (0.1% formic acid). Subsequently, 5 μL of the sample was separated by a reverse-phase chromatography using a C18 column (1.6 μm, 250 mm × 75 μm, IonOpticks, Melbourne, Victoria, Australia). Finally, the online LC-MS/MS analysis was performed with a quadrupole time-of-flight mass spectrometer. The column flow rate was 0.2 μL/min. The linear gradient profile for the standard protein separation was as follows: 3−28% phase B (acetonitrile with 0.1% formic acid) over 0−50 min, 28−45% B over 50−55 min, 45−80% B over 55−57 min, and 80% B for 3 min.

Survey full-scan mass spectra were acquired across the mass range of 100−1700 *m*/*z* in positive electrospray mode, and the accumulation and ramp times were set at 100 ms each. The single cycle acquisition period was 1.16 s, including one full TIMS MS scan and 10 parallel accumulation-serial fragmentation (PASEF) MS/MS scans. During the PASEF MS/MS scans, the collision energy increased linearly as a function of mobility, from 20 eV at 1/*k*_0_ = 0.6 V·s/cm^2^ to 59 eV at 1/*k*_0_ = 1.6 V·s/cm^2^. Other mass spectrometry parameters were set as follows: intensity threshold, 5 000; 1/*k*_0_ range, 0.75−1.35 V·s/cm^2^; capillary voltage, 1500 V; auxiliary gas flow rate, 3 L/min; ionization temperature, 200 ℃; column temperature, 50 ℃.

### Label-free quantification

The MS data were searched against the Swiss-Prot database for *Culex quinquefasciatus* (downloaded on September 6, 2024; containing 18868 protein sequence entries) using PEAKS Online for peptide and protein identifications. Search parameters were as follows: precursor mass error tolerance, 20 ppm; fragment mass error tolerance, 0.05 Da; maximum of two missed cleavages for trypsin digestion; and peptide length restricted to 6–45 amino acids. The search included variable modifications of methionine oxidation and N-terminal acetylation, and a fixed modification of carbamidomethyl cysteine. The false discovery rate (FDR) was set at 1% at both the peptide and protein group levels.

### Functional enrichment analysis

Specific antigen proteins screened by the positive antibodies in comparison to the negative antibodies were subjected to interaction network analysis using the STRING database, followed by Gene Ontology (GO) and Kyoto Encyclopedia of Genes and Genomes (KEGG) functional enrichment analyses.

### Statistical analysis

Results were analyzed using ImageJ software. Statistical analysis was performed using GraphPad Prism (version 8.0). Statistical significance was determined by one-way analysis of variance (ANOVA) followed by Duncan's multiple range test. *P* < 0.05 was considered statistically significant.

## Results

### Generation of anti-*Culex* mosquito antibodies

**Figure 1:**
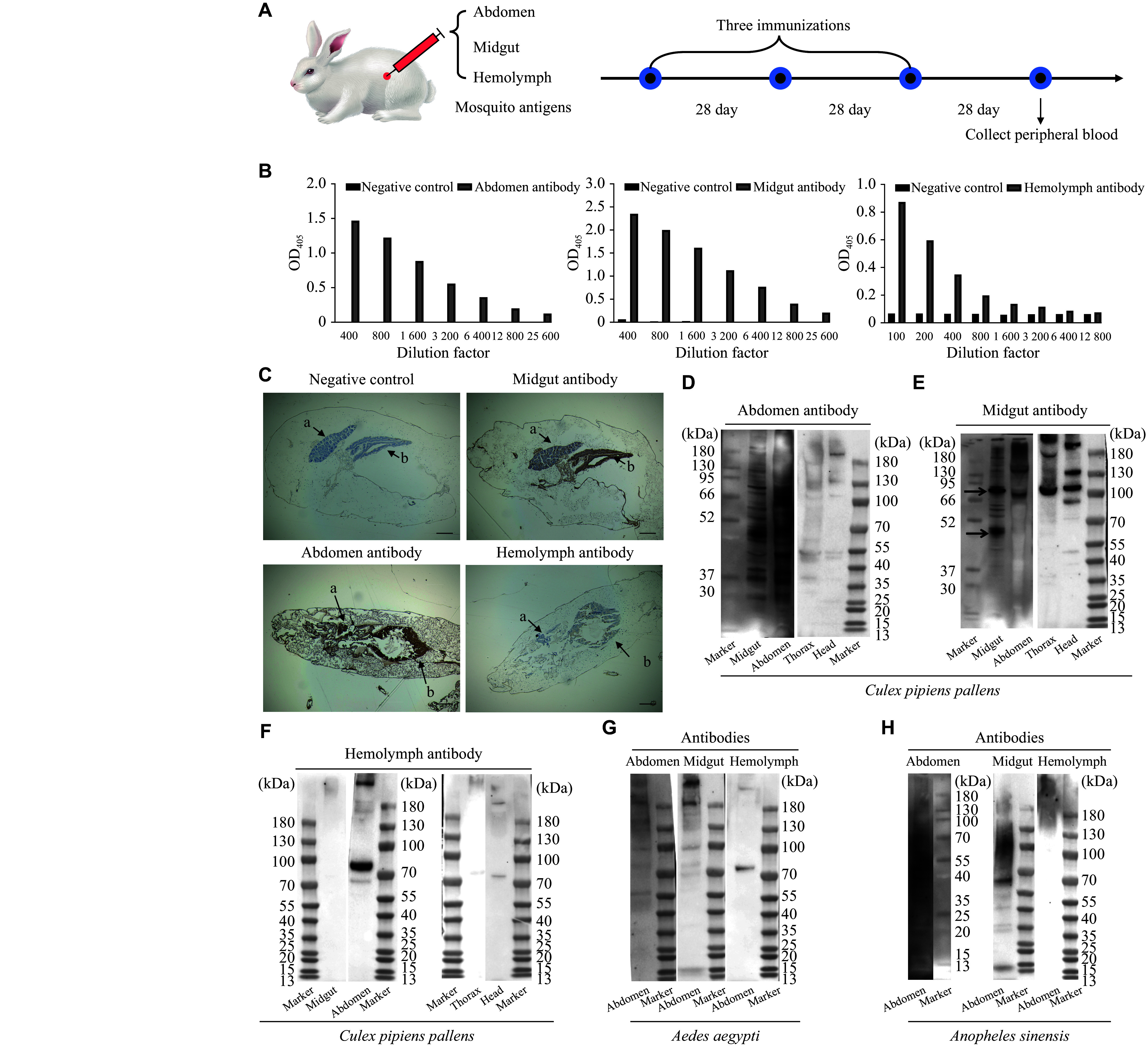
Generation of anti-*Culex* mosquito antibodies and detection of their binding ability to mosquito proteins. A: Rabbits were immunized three times at 28-day intervals with protein extracts from the whole abdomen, midgut, and hemolymph of *Culex pipiens*, using PBS as the negative control. Peripheral blood was collected 28 days after the final immunization to prepare antibodies. B: Enzyme-linked immunosorbent assay was performed to detect the binding ability of whole-abdomen, midgut, and hemolymph antibodies to corresponding *C. pipiens* abdominal antigens. C: Immunohistochemical staining was performed to examine the tissue localization of abdominal, midgut, and hemolymph antibodies in *C. pipiens* (*n* = 3 per group). a: ovary; b: midgut. Scale bar, 200 μm. D–H: Western blotting analyses assessed the binding profiles of whole-abdomen, midgut, and hemolymph antibodies against proteins from the whole abdomen, midgut, thorax, and head of *Culex pipiens* (D–F) and *Aedes aegypti* and *Anopheles sinensis* (G and H).

The immunization antigens were prepared from the whole abdomen, midgut, and hemolymph tissues of *C. pipiens*. As shown in ***Fig. 1A***, these tissue extracts were used to immunize New Zealand White rabbits (*Oryctolagus cuniculus*) in three rounds administered at 28-day intervals. This protocol successfully elicited polyclonal antibodies, which were subsequently validated by further experiments.

The binding ability of the antibodies was assessed by ELISA (***Fig. 1B***). These antibodies exhibited high titers and effectively bound to their respective antigens. For whole abdomen-specific antibodies, as the dilution factor increased (1∶400 to 1∶25 600), OD_405_ values gradually decreased, yet remained higher than those of the negative control even at higher dilutions, indicating strong binding to abdominal antigens. Midgut antibodies exhibited a dose-dependent binding pattern, with OD_405_ values at higher dilutions remaining higher than those of the negative control, indicating effective midgut antigen recognition. Although hemolymph antibodies exhibited weak binding across all dilutions, they were distinguishable from the negative control at lower dilutions, suggesting specific but weaker interaction with hemolymph antigens.

Immunohistochemical staining further revealed distinct tissue-localization patterns. Antibodies raised against abdominal and midgut protein extracts predominantly labeled the midgut and ovarian tissues of mosquitoes. Conversely, hemolymph-specific antibodies showed no detectable binding to these tissues (***Fig. 1C***). WB analysis demonstrated that both abdominal and midgut antibodies bound to their respective antigens, with major protein bands observed in the 37–95 kDa range. Notably, midgut antibodies exhibited binding to protein extracts from mosquito head and thorax tissues (***Fig. 1D*** and ***1E***). In contrast, hemolymph-specific antibodies showed binding only to abdominal protein extracts (***Fig. 1F***).

To assess cross-species reactivity, polyclonal anti-*Culex* antibodies were tested against proteins from other mosquito species. WB analysis revealed that anti-*Culex* abdomen and midgut antibodies bound to the abdominal proteins of both *Aedes aegypti* and *Anopheles sinensis*. Notably, abdominal antibodies exhibited multiple cross-reactive bands in the 35–130 kDa range in both species. Midgut antibodies also demonstrated cross-binding, particularly targeting proteins of 52–95 kDa in *Aedes aegypti* and *Anopheles sinensis* abdominal extracts. These results demonstrate substantial cross-reactive binding of *Culex*-specific antibodies to other mosquito species.

### Effects of antibodies on mosquito survival and reproduction

**Figure 2:**
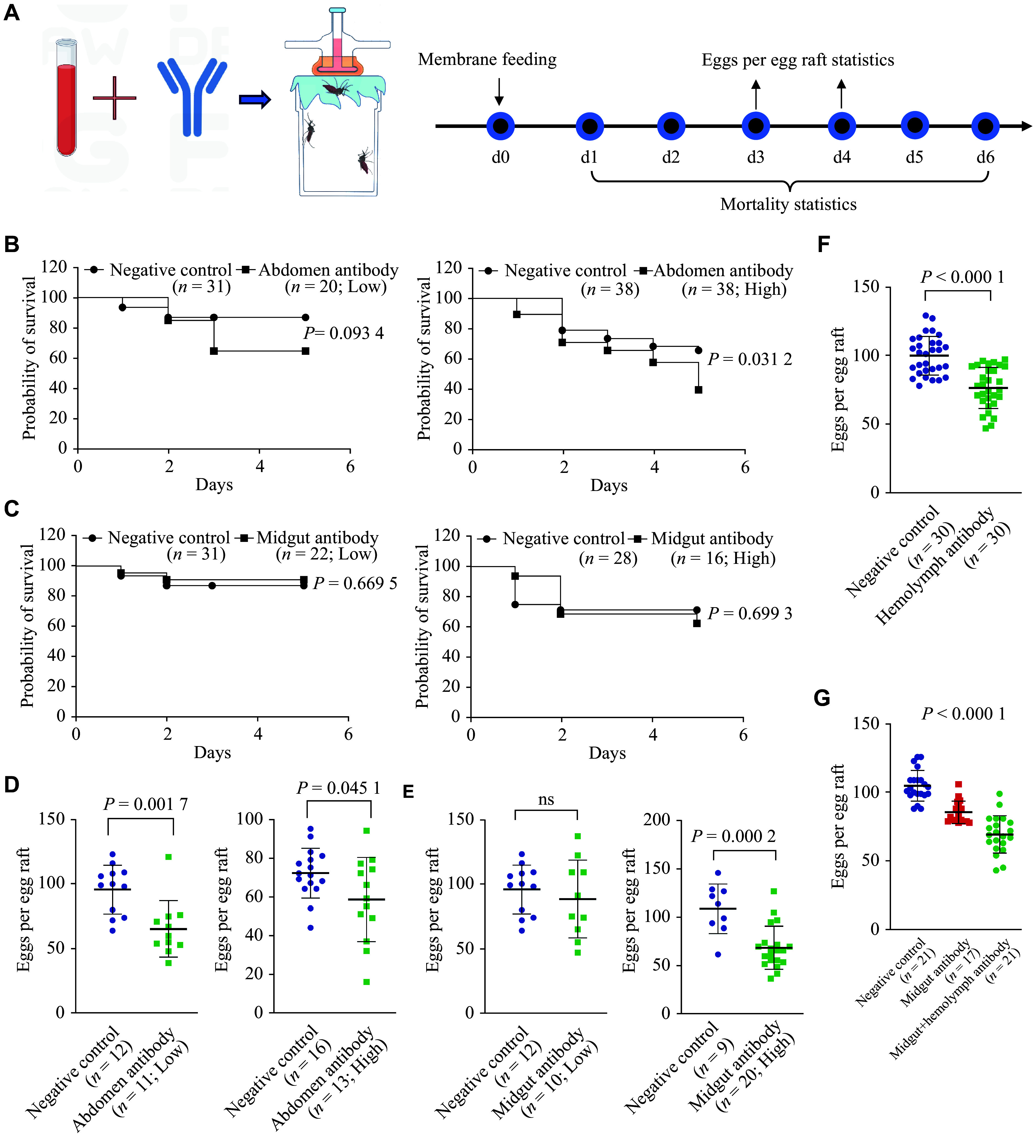
Effects of antibodies on *C. pipiens* survival and reproduction. A: Concentrated antibodies were mixed with fresh blood at ratios of 1∶9 (high concentration) and 1∶99 (low concentration) for membrane feeding. The mortality rate and egg production of *C. pipiens* were recorded within six days after membrane feeding. B and C: Mortality statistics after membrane feeding with whole-abdomen (B) and midgut (C) antibodies, respectively (Log-rank [Mantel–Cox] test). D and E: Egg production statistics after membrane feeding with whole-abdomen (D) and midgut (E) antibodies, respectively (*t*-test). F: Mortality statistics after microinjection of hemolymph antibodies (*t*-test). G: Egg production statistics after mixed membrane feeding with midgut and hemolymph antibodies (Tukey's multiple comparisons test). Data are shown as mean ± standard deviation. Each data point in the figure represents an individual mosquito.

The *in vivo* activity of polyclonal antibodies was tested using membrane-feeding assays (***Fig. 2A***). Mosquito mortality significantly increased when they were fed a blood meal containing a high concentration of whole-abdomen protein-specific antibodies, but no significant effect was observed at the lower concentration (***Fig. 2B***). Oviposition was also significantly reduced upon ingestion of a blood meal containing abdominal protein-specific antibodies; however, this effect was not further enhanced by increasing the whole abdomen protein-specific antibody concentration (***Fig. 2D***).

As the midgut is the first organ in contact with the blood meal, and the hemolymph is critical for the mosquito's physiological function, we explored whether they can be targeted by the host antibodies from the blood meal, resulting in compromised mosquito fitness and reproductive capacity. The results showed that the midgut protein-specific antibodies significantly reduced egg production at higher concentrations, but not at lower concentrations (***Fig. 2E***). Interestingly, the inhibition of egg production was further enhanced when the midgut antibodies were combined with the hemolymph-specific antibodies (***Fig. 2F*** and ***2G***). No significant effect on mosquito mortality was observed when the mosquitoes were fed with a blood meal containing the midgut-specific antibodies (***Fig. 2C***) or hemolymph-specific antibodies (Data not shown).

### Mosquitoes developed resistance to antibody-mediated oviposition inhibition

**Figure 3:**
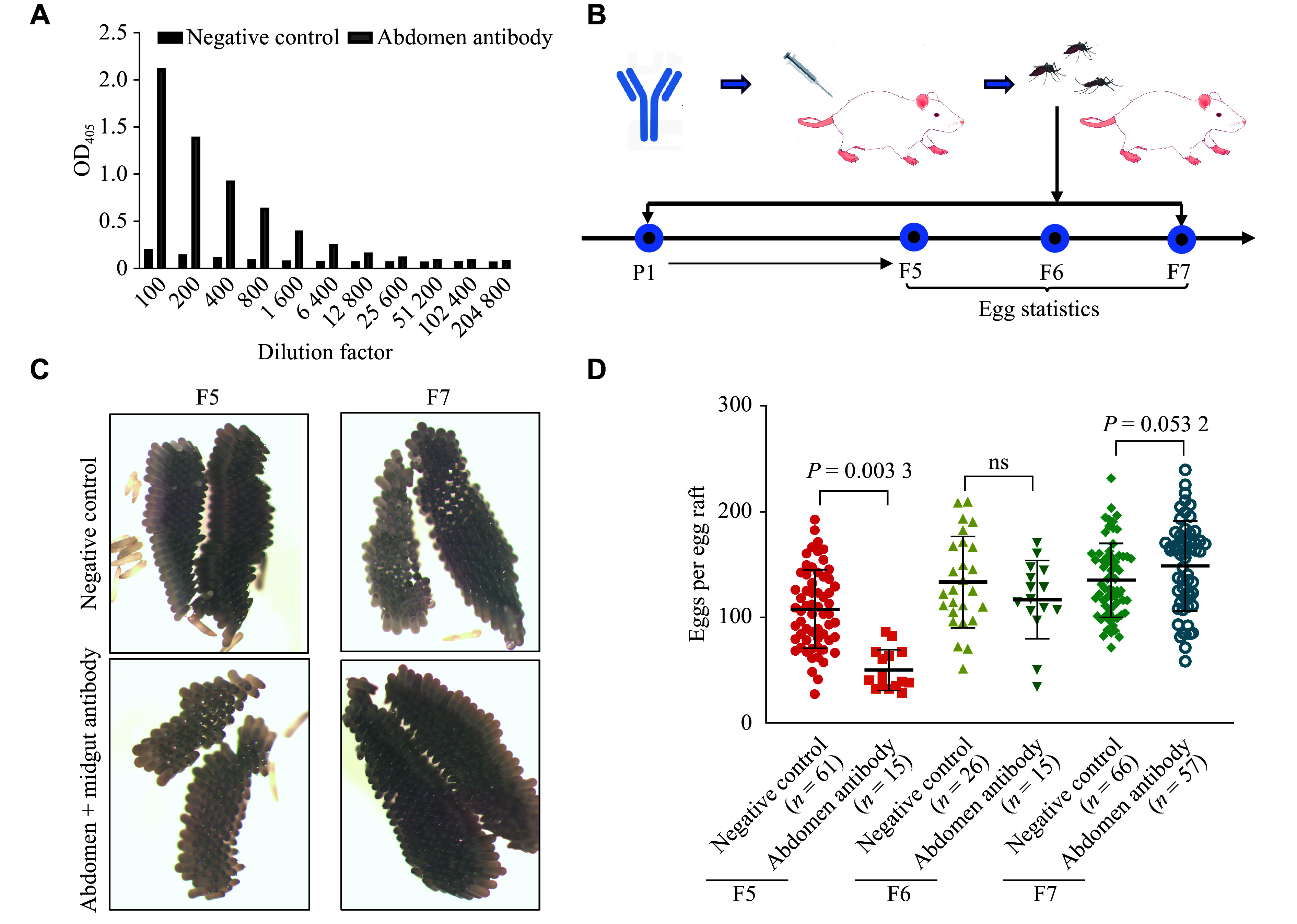
Effects of antibodies on the reproduction of *C. pipiens* offspring. A: Mouse-derived whole abdomen-specific antibodies against *Culex pipiens* were prepared using a protocol adapted from the rabbit polyclonal antibody method described in this study. The antigen-binding ability of the prepared antibodies was detected by enzyme-linked immunosorbent assay. B: The obtained whole abdomen-specific antibodies were reinfused into negative mice *via* tail vein injection. These mice were used for the propagation of *C. pipiens*, and the egg production from the F5 to F7 generations was recorded. C and D: Representative images of individual egg rafts under white light (C) and quantification of the number of eggs per egg raft (D). Data are shown as mean ± standard deviation, and were compared using the *t*-test. Each data point in the figure represents an individual mosquito.

We generated polyclonal antibodies in mice to explore the long-term effects of antibody-mediated interference. The obtained IgG antibodies exhibited high titers and effectively bound to the abdomen antigen (***Fig. 3A***). We then passively immunized mice with mosquito whole abdomen-specific antibodies to provide the blood meal for a small colony of *C. pipiens* (***Fig. 3B***)*.* A significant reduction in egg production was observed during early passages, whereas the oviposition-inhibitory effect was diminished after repeated passages (***Fig. 3C*** and ***3D***), suggesting the potential development of resistance.

### Mechanisms of antibody-mediated impairment of mosquito survival and reproduction

**Figure 4:**
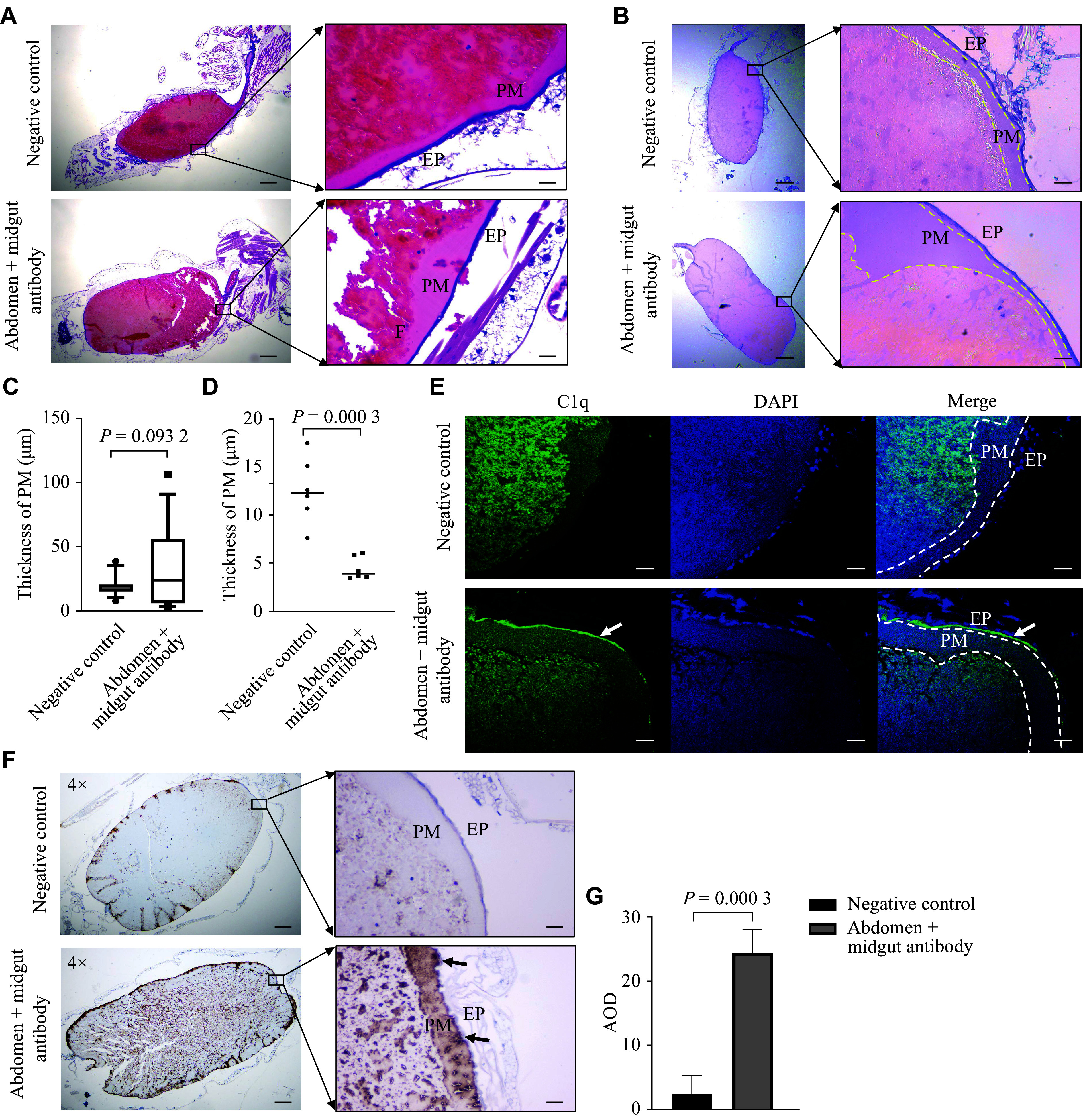
Detection of antibody-mediated midgut damage and complement activation. A: Hematoxylin and eosin (H&E) staining of midgut sections from mosquitoes fed antibody-containing blood meals (*n* = 3 for each group). Scale bar, 200 μm (left) and 40 μm (right). B: Periodic acid–Schiff (PAS) staining to visualize PM in the midgut after membrane feeding with antibodies (*n* = 5 for each group). Scale bar, 200 μm (left) and 40 μm (right). C: Quantification of PM thickness measured randomly across six regions in each cross-sectional slice of the mosquito midgut (*n* = 3). D: Quantification of the thinnest PM region in ***Fig. 4B***. E: Immunofluorescence staining to detect the complement protein C1q distribution in the midgut after membrane feeding (*n* = 3 for each group). Scale bar, 40 μm. White arrows indicate C1q bound at the midgut epithelium. F and G: Immunohistochemical staining to detect membrane attack complexes in the midgut after membrane feeding (F) and quantification based on immunostaining intensity (G) (*n* = 3 for each group). Scale bar, 200 μm (left) and 40 μm (right). Black arrows indicate the membrane attack complex formed at the midgut epithelium. Data are shown as mean ± standard deviation, and were compared using the *t*-test. Abbreviations: AOD, average optical density; EP, epithelium; PM, peritrophic membrane.

To explore the mechanisms underlying the observed effects, we first performed histological analyses on mosquitoes that ingested blood meals containing anti-abdominal protein antibodies. H&E staining of midgut sections collected after feeding showed intact midgut epithelial cells with no significant histological alterations. The blood meal was encapsulated by the peritrophic membrane, and no hemocyte accumulation was observed along the midgut epithelium (***Fig. 4A***).

Next, we examined the peritrophic matrix (PM), a crucial structure for normal digestion and mosquito survival using PAS staining^[13]^. Mosquitoes that ingested antibody-containing blood meals exhibited increased PM thickness compared with the control group (***Fig. 4B*** and ***4C***). However, as shown in the box plot, the antibody-fed group exhibited greater variability in PM thickness (***Fig. 4C***). Notably, the minimum PM thickness observed in the antibody-fed group was lower than that in the negative control group (***Fig. 4D***), suggesting potential structural weak points that may impair PM function in these regions.

Immunofluorescence staining revealed that complement component C1q was specifically localized to the midgut epithelium after mosquitoes ingested antibody-containing blood meals (***Fig. 4E***). Moreover, immunohistochemical analysis revealed a significant accumulation of complement component C9 on the midgut epithelium. These findings indicate the formation of membrane attack complexes on the mosquito midgut epithelium as a result of complement activation (***Fig. 4F*** and ***4G***).

### Targets of mosquito-specific antibodies

**Figure 5:**
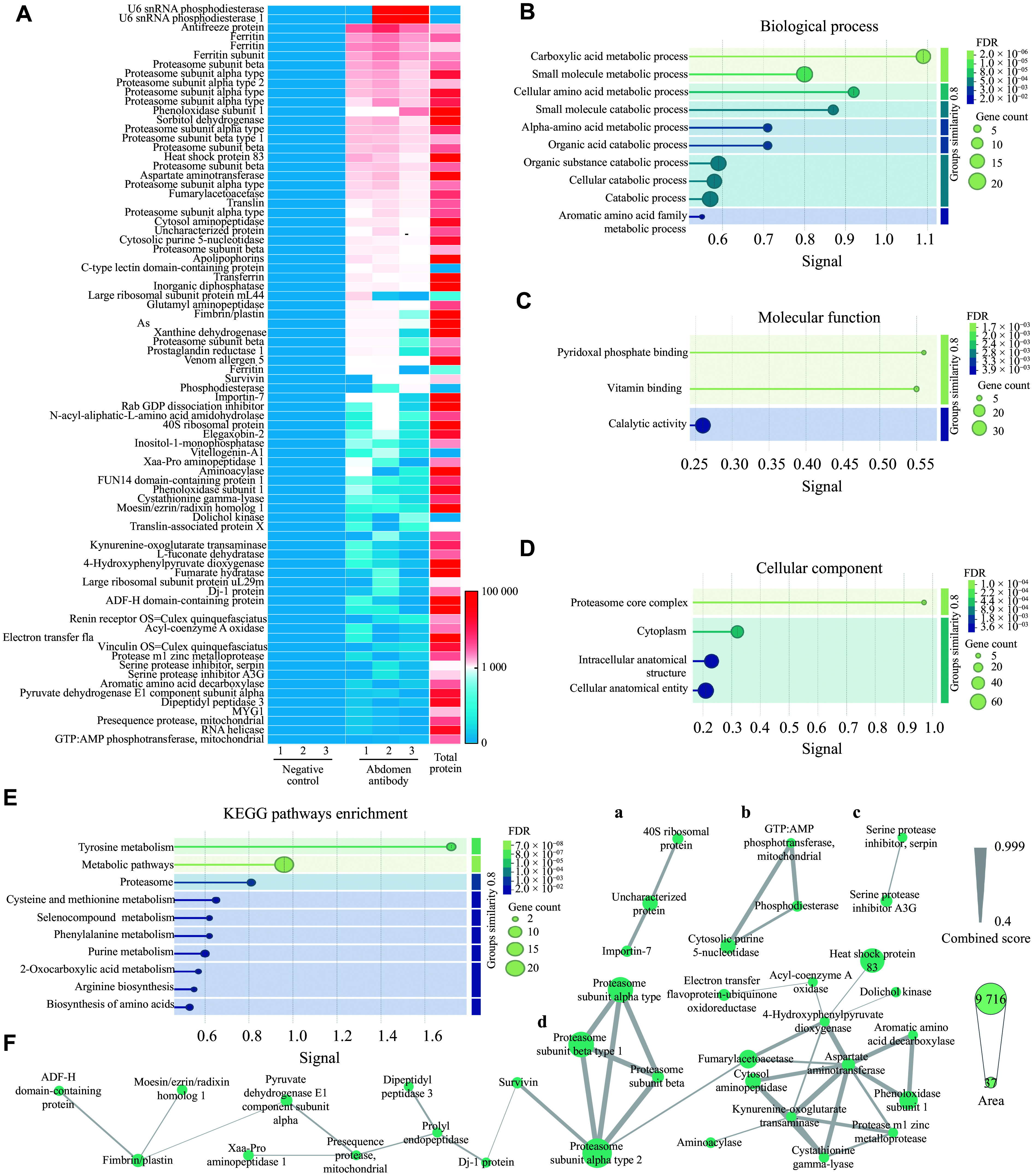
Identification of target proteins and functional enrichment analysis of *C. pipiens*-specific antibodies. A: Heatmap showing target proteins recognized by *Culex pipiens* whole abdomen-specific antibodies, compared with negative control antibodies. B–D: Gene Ontology (GO) functional enrichment analysis of target proteins, including biological processes (B), molecular functions (C), and cellular components (D). E: Kyoto Encyclopedia of Genes and Genomes (KEGG) pathway enrichment analysis of target proteins. F: Target protein regulatory network constructed based on protein-protein interactions. Node size indicates relative protein content, and line width between nodes reflects interaction strength. Panels A–D represent four groups of identified candidate target proteins with interactions, respectively.

We performed Co-IP assays to capture proteins that interact with the generated polyclonal antibodies. The proteins eluted from the Co-IP experiments were then analyzed by LC-MS/MS. Ultimately, a total of 80 proteins were identified with significant enrichment relative to the background (***Fig. 5A***). To characterize their functions, GO enrichment analysis was performed. These proteins were assigned to biological processes, cellular components, and molecular functions. The most significantly enriched GO terms were carboxylic acid metabolic process, small molecule metabolic process, cellular amino acid metabolic process, vitamin binding, pyridoxal phosphate binding, catalytic activity, proteasome core complex, and cytoplasm (***Fig. 5B***–***5D***). The enriched GO terms were predominantly associated with metabolic processes. Based on KEGG enrichment analysis, the proteins were also enriched in pathways related to metabolic functions, including metabolic pathways, tyrosine metabolism, proteasome, purine metabolism, cysteine and methionine metabolism, phenylalanine metabolism, and selenocompound metabolism pathways (***Fig. 5E***). Four co-expression networks clustered multiple proteins into functional modules based on their abundance correlations derived from mass spectrometry (***Fig. 5F***).

In the network analysis, several key proteins with strong interactions and high enrichment were identified. These included 4-hydroxyphenylpyruvate dioxygenase, aromatic amino acid decarboxylase, aspartate aminotransferase, cystathionine gamma-lyase, cytosolic aminopeptidase, fumarylacetoacetase, kynurenine-oxoglutarate transaminase, phenoloxidase subunit 1, protease M1 zinc metalloprotease, and proteasome subunit family proteins, which served as hub proteins in the interaction network.

Because antibodies ingested in the blood meal cannot access intracellular compartments, we restricted our analysis to membrane-associated proteins and secreted proteins. A total of six membrane-associated proteins and six secreted proteins were identified with significant differences relative to the background. The identified proteins are listed in ***Table 1***.

**Table 1:** The list of candidate membrane and secretory proteins

Protein types	Accession	Peptides	Average mass (kDa)	Description
Membrane proteins	B0W1Y3	20	113.26	Protease M1 zinc metalloprotease
Membrane proteins	B0WS53	3	34.99	Renin receptor
Membrane proteins	B0WVN6	31	116.56	Glutamyl aminopeptidase
Membrane proteins	B0WYY2	52	67.75	Moesin/ezrin/radixin homolog 1
Membrane proteins	B0X394	32	107.16	Vinculin
Membrane proteins	B0X886	27	69.60	Transferrin
Secretory proteins	B0WAG6	20	366.98	Ferritin-1
Secretory proteins	B0WKS6	157	24.55	Apolipophorins
Secretory proteins	B0X3X9	6	28.98	Ferritin-2
Secretory proteins	B0X6A5	11	69.60	Venom allergen 5
Secretory proteins	B0X886	27	69.60	Transferrin
Secretory proteins	B0XAL1	7	46.09	Serine protease inhibitor A3G

## Discussion

Our study provides proof-of-concept evidence for the feasibility of harnessing host immune responses to impair mosquito survival and reproductive fitness by immunizing hosts with mosquito antigens to generate mosquito-specific antibodies. Notably, we identified complement activation and membrane attack complex formation as key mechanisms underlying antibody-induced mosquito mortality and reproductive impairment, and pinpointed potential targets for this effect. These findings highlight the potential of utilizing antibody-complement interactions as a targeted strategy to suppress mosquito populations.

Using antibodies targeting *C. pipiens* abdominal proteins, we observed significant reductions in egg production and increased mortality, especially at higher concentrations. This result is consistent with previous reports^[8]^. However, the whole abdomen-specific antibodies consisted of a mixture of antibodies recognizing multiple tissue targets, making it difficult to elucidate the precise mechanisms involved. These mechanisms were further explored by feeding the mosquitoes with antibodies specific to the midgut and hemolymph. Our data indicated a weak but dose-dependent inhibition of oviposition mediated by midgut-specific antibodies, which was further enhanced by the addition of hemolymph-specific antibodies. A possible explanation is that midgut integrity was initially compromised by midgut-specific antibodies, potentially causing structural damage or increased permeability of the midgut epithelium, after which mosquito fitness was further impaired when hemolymph-specific antibodies that leaked through the compromised gut barrier disrupted hemolymph function. These findings emphasize the importance of midgut integrity in mosquito fitness and suggest that targeting midgut proteins could represent an effective strategy for vector control.

Our hypothesis was supported by histological and immunohistochemical analyses, which demonstrated that midgut-specific antibodies triggered significant complement activation, leading to damage to midgut epithelial cells and increased gut permeability, thereby potentially compromising mosquito physiological homeostasis and reducing reproductive success. These findings are consistent with previously published studies suggesting that complement activity can impair the mosquito metabolic processes^[14]^.

Metabolic processes play a central role in the life and reproductive processes of mosquitoes, not only providing energy and nutrition for their survival and growth, but also affecting their reproductive ability and success rate by regulating hormone levels and metabolites^[15]^. Although our antibodies target the midgut and abdominal segments of mosquitoes, the targeted proteins are mostly enriched in metabolism-related GO terms and KEGG pathways, suggesting that antibodies may affect mosquito reproductive ability by impairing their metabolism. For example, inhibiting the metabolism of phenylalanine results in reduced oviposition, delayed vitellogenesis, and impaired chorion melanization in mosquitoes, thereby leading to a significant decline in their reproductive capacity^[16]^. However, antibodies delivered through the blood meal are unlikely to bind to intracellular antigens. Therefore, membrane-associated or secreted proteins such as transferrin and ferritin have attracted our attention. Previous studies have shown that inhibiting the function of transferrin can significantly reduce the reproductive capacity of female mosquitoes^[17]^. Additionally, ferritin-based nanoparticle vaccines have shown effective and long-lasting activity in reducing the spread of malaria^[18–20]^. These results indirectly suggest the reliability of protein screening for anti-mosquito key targets based on whole abdominal and midgut antibodies by immunoprecipitation. Similarly, the metabolic status of the mosquito is crucial for *Plasmodium* development^[21]^. Thus, the proteins we have screened are expected to help further explore their role in inhibiting the spread of mosquito-borne pathogens. While our study provides compelling evidence for the efficacy of mosquito-targeted immunization strategies, several challenges remain. The variability in antibody responses across different mosquito species, the potential for immune evasion, and the need for scalable vaccine formulations require further investigation. Additionally, the ecological influence of reducing mosquito populations through immune-based interventions should be carefully assessed to ensure sustainability and minimize unintended consequences.

In conclusion, our findings establish a foundation for the rational design and development of mosquito-targeted vaccines as a novel vector control strategy. By demonstrating the feasibility of antibody-mediated suppression of mosquito survival and reproduction and by identifying key molecular targets, our study lays a foundation for future research aimed at developing an optimized vaccine formula through reverse vaccinology and assessing its real-world applicability in reducing target mosquito populations in natural settings. With continued advancements in vector immunology and vaccine technology, the prospect of an effective, species-transcending mosquito vaccine holds significant promise for alleviating the global burden of mosquito-borne diseases.
